# Patient and Health Professional Perceptions of the Assessment, Diagnosis and Management of Acute Charcot Neuro‐Osteoarthropathy at a Regional Australian Health Service

**DOI:** 10.1002/jfa2.70079

**Published:** 2025-09-06

**Authors:** Dimitri Diacogiorgis, Byron M. Perrin, Emma MacDonald, Michael I. C. Kingsley

**Affiliations:** ^1^ Department of Allied Health Assistants Grampians Health Ballarat Australia; ^2^ La Trobe Rural Health School La Trobe University Bendigo Australia; ^3^ Holsworth Research Initiative La Trobe Rural Health School La Trobe University Bendigo Australia; ^4^ Allied Health Education and Research Unit Goulburn Valley Health Shepparton Australia; ^5^ Department of Exercise Sciences Faculty of Science University of Auckland Auckland New Zealand

**Keywords:** charcot, diabetes, foot, neuroarthropathy, peripheral neuropathy

## Abstract

**Background:**

Acute Charcot neuroarthropathy (CN) is a rare but serious complication of diabetes that requires timely diagnosis and evidence‐based management to prevent long‐term disability. In regional or rural settings, delivering evidence‐based care is particularly challenging due to systemic and contextual barriers.

**Objective:**

To explore the perceptions of patients and health professionals about assessment, diagnosis and management of acute CN in a regional Victorian health service.

**Method:**

This study used a qualitative research design, utilising thematic analysis of semi‐structured interviews with patients with previous acute CN and focus groups with health professionals (orthopaedic surgeons, podiatrists and prosthetists and orthotists) involved in the assessment and management of patients with acute CN. Two assessors used inductive thematic analysis to identify key themes related to acute CN care delivery.

**Results:**

Four overarching themes were identified: (1) barriers to evidence‐based care, including delayed diagnosis, limited access to skilled clinicians and diagnostic tools and the burden of treatment; (2) enablers, such as timely access to knowledgeable clinicians and resources; (3) mitigating factors, including patient engagement, empathetic communication and multidisciplinary support and (4) strategies for improvement, such as public and professional education, upskilling of health professionals and integration of psychological and person‐centred support.

**Conclusion:**

Improving outcomes for people with acute CN in regional or rural settings requires a multifaceted approach. Enhancing awareness, building workforce capacity and embedding patient‐centred care practices are essential to ensure timely diagnosis, equitable access to treatment and improved quality of life.

**Abbreviations**
CN: Charcot Neuro‐osteoarthropathy
HRFC: High Risk Foot Clinic
MRI: Magnetic Resonance Imaging
TCC: Total Contact Cast

## 1 Introduction

Acute Charcot neuro‐osteoarthropathy (CN) is characterised by an inflammatory process in the presence of peripheral neuropathy that if managed suboptimally can have a disastrous health sequelae impacting physical and mental well‐being [1, 2, 3, 4]. Clinical guidelines provide recommendations on the care of people with acute CN but these are based on limited evidence [2, 5]. Timely offloading or immobilisation of the affected foot is critical to reducing the swelling, heat and redness associated with inflammatory process and to avoid serious complications such as structural deformity of the foot, skin ulceration, infection and lower limb amputation [5, 6, 7]. This is achieved initially through total contact casting (TCC) which is a fibreglass custom moulded irremovable knee high cast which is removed and reapplied regularly [2, 8]. Early access to a multidisciplinary high risk foot clinic (HRFC) can assist the timely assessment and diagnosis and management of acute CN [9].

However, there are significant challenges to timely access to care, which can be exacerbated for those who reside in regional or rural settings [10, 11, 12, 13]. The management process of acute CN can also be lengthy, and quality of life and the ability to undertake activities of daily living is negatively impacted during and in the long term [3, 4, 14]. In a regional/rural setting, there is likely to be less skilled health professionals available to identify, diagnose and manage acute CN locally [11, 12, 13]. Availability of resource intensive gold standard diagnostic and monitoring technology might not be readily available [10]. The current evidence base is largely reflective of tertiary level health services practice [10]. As such, there are limited practical guidelines on how to best implement the care of patients with acute CN particularly in a regional or rural setting [15, 16].

A better understanding of the regional/rural context in relation to the provision of the care of acute CN is needed. Therefore, the aim of this study was to explore patient and health professional perceptions of the delivery of the assessment, diagnosis and management of acute CN in a regional Victorian health service health catchment area.

## 2 Method

### 2.1 Study Design

This study used thematic analyses of semi‐structured interviews for patients with previous acute CN and focus groups with health professionals involved in the assessment, diagnosis and management of patients with acute CN. Ethical approval was granted by the Grampians Health Human Research Ethics Committee (ref 90034) and the La Trobe University Human Research Ethics Committee (ref 90034).

### 2.2 Setting

The Grampians, a regional area of Victoria, Australia has a population of over 250,000 people, comprises 47,980 square kilometres, representing 20% of Victoria's land mass [17]. The Grampians region of Victoria incorporates areas classified from larger regional (MMM2) to smaller rural centres (MMM5) according to the Modified Monash Model [18]. Grampians Health is the main public health service in this region.

People with acute CN travel to Ballarat to attend the Grampians Health HRFC. The HRFC incorporates orthopaedic, infectious diseases, endocrinology and vascular specialists along with allied health, predominately podiatry and prosthetics and orthotics. Clinical sessions run most days of the working week (i.e., Monday to Friday) to enable prompt access for those who have high risk foot conditions requiring urgent care. Patients with acute CN who are referred to the HRFC are offered the same or next working day review.

### 2.3 Participant Recruitment

Australian Health Practitioner Regulation Agency (AHPRA) registered orthopaedic surgeons and podiatrists and Australian Orthotic Prosthetic Association (AOPA) credentialled prosthetic and orthotic staff at Grampians Health (GH) were invited to participate in face‐to‐face focus groups by letter via in person verbal enquiry, email or post. These three professional groups were invited to three separate focus groups due to their critical involvement in the care process for patients with acute CN.

Patients 18 years of age or older with prior acute CN who presented to GH between 2017 and 2019 were invited to participate in semi‐structured interviews by email, post or via verbal in‐person enquiry. Participants were reidentified from a previous retrospective cohort study of patients treated for acute CN at Grampians Health [11]. The focus groups and structured interviews were facilitated by the first author who is a senior podiatrist at Grampians Health. All participants provided written informed consent prior to participation in the study.

### 2.4 Data Collection

#### 2.4.1 Focus Groups

All focus groups with health professionals were conducted at Grampians Health Ballarat. The focus groups were guided by the semi‐structured question schedule (Table 1). Focus group questions were developed with reference to a systematic review and retrospective cohort study [10, 11]. These studies identified potential enablers and barriers to best practice care such as patient knowledge of acute CN, timely access to care and availability of diagnostic imaging. To gain a deeper understanding of the health professionals' perceptions of acute CN, general questions relating to enablers and barriers to best practice care in the region were also posed.

**TABLE 1:** Focus group questions.

Number	Question
1	From your experience and perspective, can you describe some of the enablers and barriers to care of the person with acute Charcot neuroarthropathy?
2	Our local data suggest that just under 50% of patients with Charcot neuroarthropathy did not know when or how their foot was injured. How does this finding relate to your experience of working with patients with acute Charcot neuroarthropathy?
3	Our local data suggest that most patients with acute Charcot neuroarthropathy were diagnosed and then referred into Grampians health by external providers. What are some of the factors that may impact time to treatment in this scenario?
4	What would be your preferred method of diagnostic imaging to diagnose and monitor acute Charcot neuroarthropathy and why?
5	What would be your approach to the treatment of a patient with acute Charcot neuroarthropathy?

#### 2.4.2 Structured Interviews

Structured interviews with patients were conducted by phone or face‐to‐face at Grampians Health Ballarat. One‐to‐one interviews were conducted to provide the participant space and time to recount their experiences. As previous studies have identified a general patient lack of understanding of acute CN, the questions asked aimed to allow the participant to articulate their experience and perceptions of acute CN in their own preferred way. The three broad questions sought to prompt the participants to recount their experience of acute CN management across the care continuum (Table 2).

**TABLE 2:** Structured interview questions.

Number	Question
1	Can you describe your journey and experience of having Charcot foot?
2	How were you diagnosed with Charcot foot and what happened once you had this diagnosis?
3	What was your experience in the treatment of your Charcot foot?

### 2.5 Data Transcription and Analysis

The focus groups and structured interviews were audio‐recorded using the Voice Memos application on a handheld mobile device (Iphone Model X; Apple Incorporated, United States). Audio data were then transcribed verbatim into a word document (Microsoft Teams; Microsoft Corporation, United States). The focus group and structured interview recordings remained uninterrupted while being conducted.

All focus group and structured interview transcripts were imported to the qualitative data analysis computer software (NVivo for Mac; Lumivero, United States), and each transcript was analysed separately by participant type (health professional or patient). Inductive thematic analysis was used to allow for meaning and the identification of themes to occur organically [19]. Coding was undertaken by two authors (DD and EM) to identify themes in the transcripts. The authors independently undertook initial broad brush coding, reflection and further thematic coding to determine the emerging key domains. Coding was then compared and discussed to consolidate understanding of how the data represent these key domains to determine the final themes. Disagreements were resolved through discussion to reach consensus on the key themes [19].

## 3 Results

Nine structured interviews (range 15–42 min) were undertaken with patients with acute CN who presented to Grampians Health for assessment, diagnosis and management between 2017 and 2019 (67% female, 58.2 ± 7.8 years). Three focus groups (range 26–36 min) were conducted with three orthopaedic surgeons (18.3 ± 11.6 years experience), five podiatrists (5.4 ± 3.5 years experience) and 5 prosthetists/orthotists (10.2 ± 7.6 years experience).

Four overarching themes were identified by both the health professionals and the patients (Table 3): Theme 1: Barriers to evidence‐based assessment, diagnosis and management of acute CN; Theme 2: Enablers to the evidence‐based assessment, diagnosis and management of acute CN; Theme 3: Mitigating factors for the negative impact of Charcot management and Theme 4: Suggestions for strategies to improve the care of acute CN.

**TABLE 3:** Supplementary quotes relating to four main themes.

Health professional	Patient
Barriers: Barriers to evidence based assessment/diagnosis/management	
Inadequate knowledge/insight	
Health professional knowledge gaps: *‘I suppose external providers knowing about the services…They may not know that there is High Risk Foot Service in the region’ (Podiatrist participant 3)* *‘Sometimes it goes back to the GP. I know there has been a couple of examples…where GPs diagnosed it as Gout and didn't know why it wasn't improving … some GPs aren't even aware of Charcot …’* *(Prosthetist*/*Orthotist participant 3)*	Health professional knowledge gaps: *‘Surgeon who amputated the toe …‘says, oh … I see you spent four days in the emergency department.’ and I said ‘Yeah, what was that about?’ He said ‘Oh, they overreacted.’ I thought to myself I just sold my business and my livelihood’* *(Patient participant 4)* *‘I was seeing all different doctors you see with all different opinions and I was getting frustrated. I was sitting outside the clinic and I heard two nurses talking saying that don't talk to XXX about his feet as he doesn't want to hear it. They were saying I might lose it and that scared me’* *(Patient participant 9)*
Patient knowledge of signs and consequences of Charcot: *‘A lack of wanting to know and understand and wishing they didn't have an issue and perhaps a little bit of ignorance that its fine it will get better and my foot will go back to what it was because it's like ‘Oh I've broken a bone I'll put a cast on that'll fix it and my foot will come out the way it was before’* *(Prosthetist/Orthotist participant 3)* *‘So someone who had it (Charcot) for the first time might not be give or accurately tell you what's wrong’* *(Prosthetist/Orthotist participant 5)* *‘ … the patient is not educated adequately on the urgency of getting an appointment with us. They may start to think as you said its cooling down I am getting better so I am ok now’* *(Podiatrist participant 4)* *‘there's still a percentage of people that just sort of don't get their head around what they need to know that … have 'Charcot brain’* *(Orthopaedic surgeon participant 3)*	Patient knowledge gaps: *‘I hadn't noticed and had not taken it all on board and that was the result I was just thinking it would get better’* *(Patient participant 16)* *‘I was quite shocked actually and I had to go home and google it (Charcot) and it was explained here of course at the high risk clinic but I still went home and did a bit of research …’* *(Patient participant 16)* *‘No, never (previously heard of Charcot). So it was a very big shock for me, yeah. It … was something completely out of the blue.’* (*Patient participant 7)*
Lack of community awareness: *‘Not many people know what it is or what to look out for … that's a problem because its not well known in the community’* *(Podiatrist participant 3)*	Lack of community awareness: *‘No matter what people say there is always bigotry out there. And people look at you … because you have this big … cast on … and then you have to go through a 10 min spiel because no one has heard of Charcot foot’* *(Patient participant 16)*
Perception of patient capacity and insight: *‘there's still a percentage of people that just sort of don't get their head around what they need to know that … have 'Charcot brain’* *(Orthopaedic surgeon participant 3)* *‘Possibly other medical concerns for the patients … they have higher priority … (over Charcot management)’* *(Podiatrist participant 1)* *‘Patients understanding due to their health literacy…I have seen people…where I have said 'you have an active Charcot I think you need to go to your GP today' Then they just didn't and have fallen out of the system somewhere’* *(Prosthetist/Orthotist participant 4)*	Patient insight: *‘So maybe some education at that early stage may have helped. I don't know because generally speaking a person is often in denial about what's going on and I think I was in denial’* *(Patient participant 20)*
Poor access	
Right professional, right skills and right time: *‘Definitely processes. I think we have a clunky system that unless they ring up and speak to a Podiatrist or P&O specifically they (referrer) just send off a referral and sometimes that can take days or even a week to get through central intake and then to us’* *(Prosthetist*/*Orthotist participant 3)* *‘Biggest barrier was getting up skilling (in Total Contact Casting) getting some education, having some maybe clinical supervision in seeing it done, you know,learning the new technique and then applying it and having some someone come and shadowt’* *(Podiatrist participant 2)* *‘I can't foresee barriers here (to applying TCC) as such. I think the biggest barrier was getting up skilling, getting some education, having some maybe clinical supervision in seeing it done … learning the new technique and then applying it and having some someone come and shadow … I think we could possibly do it here’* *(Podiatrist participant 2)*	Right professional, right skill and right time: *‘I felt angry…because I was like 4 weeks walking on it and I feel … if I'd got it in (to High Risk Foot Clinic) the first week, I don't think I'd have the bump….I honestly believe that's super disappointing.’* *(Patient participant 7)*
Financial barriers to care: *‘ … its not always clear on how they can get to the appointments..(if) … there is a funding out there for them to arrange transport … a lot of them don't have that and they are having to find their own way in which would be difficult for weekly appointments’* *(Podiatrist participant 1)* *‘ … with total contact casting you can't drive and if they don't have a carer or someone else … in their life to help them … you can't put them in a TCC’* *(Prosthetist/Orthotist participant 2)* *‘We're trying to tell them to have a TCC or this is a long treatment or long treatment process and at the end we need to give you these hundred dollar shoes and expensive devices they're going to be like “Well why can't I go back to my Kmart shoes?”’* *(Prosthetist*/*Orthotist participant 3)*	Financial barriers to care: *‘… he said that I needed to have interventive surgery straight away and I explained to him I didn't have the type of health insurance or the funds…and that's when he said you know you have to go through the public system’* *(Patient participant 16)*
Lack of access to diagnostic imaging: *‘… (we) do not have an MRI in XXXX so we need to send them away to other towns to get the imaging’* *(Podiatrist participant 2)* *‘The quickest would be an Xray but the best would be an MRI. Getting an MRI for everything is not practical sometimes.’* *(Podiatrist participant 5)*	
Burden of Charcot management	
Patient burden of engaging with Charcot management:	Patient burden of engaging with Charcot management:
Patient transport: *‘They don't have the means to come to the appointment in the first place then they think twice about it’* (*Podiatrist participant 5)* *‘… its not always clear on how they can get to the appointments. (if) … there is a funding out there for them to arrange transport so I hear that a lot of them don't have that and they are having to find their own way in which would be difficult for weekly appointments’* *(Podiatrist particpant 1)*	Patient transport: *‘Originally this started off with appointments first thing in the morning and I said well, you know like we got three hour drive.’* *(Patient participant 4)* *‘… Where I lived I had to take two buses and that was a bit of a nightmare’* *(Patient participant 7)* *‘I mean I was still very devastated cause I couldn't drive …’* *(Patient participant 16)*
Enablers: To evidence based assessment/diagnosis/management	
Timely access to skills, knowledge and resources for Charcot management	
Timely access to specialists in Charcot management: *‘I think having an early appointment to diagnose the Charcot or.rule it out seems to be one of the most important parts. If they come in a week…after the symptoms…begin the result … is more positive than for someone who comes in six weeks after noticing changes’* *(Podiatrist participant 1)*	Timely access to specialists in Charcot management: *‘I probably rang a couple of times to ask questions and you all were great and more than happy to answer questions.’* *(Patient participant 16)* *‘I came in one day when I didn't have an appointment and one of the podiatrists fixed it all up…’* *(Patient participant 10)* *‘He said there is a high risk foot clinic and he rung you straight away and I went staright down came here and started the process … I Went from seeing my doctor with a sore foot into my first cast’* *(Patient participant 16)*
Skills training for health professionals in Charcot management: *‘I think the need to upskill more staff.… the ability for more staff to help at different times…so we have some flexibility and ability to fit these patients in.’* *(Prosthetist/Orthotist particpant 3)*	Clear accurate education early in the Charcot management process: *‘I think because it (Charcot) was explained so well and it was a happy friendly team who were very informative and very caring and it made a big difference’* *(Patient participant 16)*
Access to aids and in‐house modifications to support Charcot management: *‘We use knee scooters with some of them it all depends on age of the person what does the person do’* (*Podiatry participant 5*)	Access to public funding: *‘Being a public patient I have not had to pay a bloody cent at all for it’* *(Patient participant 19)*
Charcot stage transition: *‘I think it's a combination of how much the temperature differential the other side is and how much their volume changes are between applications and total contact casts.’* *(Orthopaedic surgeon participant 1)*	Charcot stage transition: *‘It had become my friend that boot. It gave me stability. It gave me a lot of things like that because it was making me walk. I Was afraid if I took the boot off for too long that it would come back. It was like a security blanket I've really missed it actually. So unstable that foot and it becomes the boot becomes your leg’* *(Patient participant 7)*
Mitigating factors for negative impact of Charcot management	
Multidisciplinary approach to care: *‘But what works well … is the multidisciplinary approach. Having people from multiple facets see our patients in terms of access … To multiple screen team, it's important and the the thing that works well … I've noticed this patient compliance’* *(Orthopaedic surgeon participant 2)*	Patient resilience: *‘I can't really run with that foot … I can't really run but with regards to limitations there is nothing really you can't do ‐ just got to keep my shoe on that foot and keep it protected’* *(Patient participant 15)* *‘It was like no it has to be done and we just gotta do it. That was my mentality. If it has to be done it has to be done. If I had to go a few more months up to two years that I would've accepted it’* *(Patient participant 15)* *‘I've learnt or I have had to change my whole life essentially. I've learnt what I can and can't do and I probably understand more about my feet. I Think you take your feet for granted’* *(Patient participant 16)* *‘I had to get out … The rehabilitation as fast as I could to try and be independent. I Have always … tried to be as independent as possible … and that's probably one of the reasons why today I don't walk with a walking stick or a wheeler’* *(Patient participant 20)*
	Trust in the treating team: *‘Now you guys were very helpful … You did whatever was possible to help me with my situation and how I couldn't have asked for anymore’* *(Patient participant 4)* *‘The girls were so good here and the guys you did, it was never a problem. And I used to feel really bad that they had to waste so much plaster. You know, things like that is my thought, but I've never had a crossword with anyone. Never had a problem with anyone. It was just easy, so and it was almost like you needed someone to say it's OK and I got that here.’* *(Patient participant 7)*
Patient knowledge of and insight into their condition: *‘Seeing the X‐rays showing what the foot structure looks like as compared to what it should look like’* (*Prosthetist*/*Orthotist participant 3*)	Capacity for self‐expression in offloading: *‘We need different colour plaster. I Know I spoke about this all the time. The fact that you are lying on your stomach and you can't see what's happening and you turn over and you have this really lumpy weird piece of white plaster. I Think we need to change that somehow to try and lighten it for people’* *(Patient participant 16)*
	Support to maintain employment during treatment: *‘I could work … with (my employer) when I had the black boot on. I wasn't allowed to when I was in plaster’* *(Patient participant 7)*
Suggestions for improvements to Charcot management	
Improved access to diabetes screening and patient education: *‘Ensuring that diabetes educator is educated on this subject (Charcot Screening/Diagnosis) themselves would be a good start’* *(Orthopaedic surgeon participant 3)*	Public awareness and access for Charcot management: *‘Some people use pamphlets, some people listen to the radio. Most people are on their phones on some platform. You could do it (Charcot education campaign) on some media type of thing, Facebook or other thing’* *(Patient participant 10)*
Use of person‐centred motivational techniques: *‘Motivational interviewing, like seeing what this person's goals actually are, giving them the information … (to) get them to those goal’* *(Podiatrist participant 5)*	Psychological and peer support: *‘I would suggest … speaking to a psychologist …’* *(Patient participant 19)*
Education for health professionals about Charcot management: *‘If we're training staff up in the region even if they do leave they are taking that skill with them to another service who may or may not be doing TCCs’* *(Prosthetist/Orthotist participant 3)*	Education for health professionals about Charcot management: *‘I think the doctors need to know because the doctor that I saw … had no idea’* *(Patient participant 12/13)*

Subthemes were identified for each overarching theme, which showed subtle differences between the health professionals' and patients' perceptions leading to the overarching themes (Table 3).

### 3.1 Theme 1. Barriers to Evidence‐Based Assessment, Diagnosis and Management of Acute CN

#### 3.1.1 Inadequate Knowledge and Insight

Both interview and focus group participants perceived health professional knowledge gaps as negatively impacting the early identification of acute CN in the assessment and diagnosis aspects of the care pathway.

Suspecting Charcot in the first place.… then it’s been three months and its (the foot) not getting better and then they're referred to the High Risk Foot Service

(Podiatrist Participant 5)

Some respondents' experienced health professional's knowledge gaps as a misalignment in response to the presentation of CN symptoms, either in as incorrect diagnosis, therapy or incorrect prognostic advice causing distress.

The … hospital didn't know that's what my GP was gonna look for … they had the (imaging) request … (querying) Charcot … and they didn'*t* test it … they treated me for (infection)

(Patient participant 7)

Health professionals believed that limited patient knowledge was a barrier to early recognition of the symptoms of CN and others that limited insight into the seriousness of acute CN resulted in a lack of patient urgency in engaging in treatment. Health literacy and competing health priorities were also identified as factors in patient engagement with CN management.

Unfortunately, it’s inevitable they (people with CN) … get disassociated (due to neuropathy) and … it's almost like they … cease to exist below the knees … and … don't see what’s happening

(Orthopaedic Surgeon participant 1)

However, some patients reported that they had insight into their limited initial understanding of acute CN and associated treatment pathways. They reported that their ability to comprehend information during the immediate diagnostic and treatment period was impacted by shock and apprehension regarding the uncertainties pertaining to the implications of a CN diagnosis, which at times, initially resulted in denial.

After that (CN diagnosis) every single minute that’s all you hear inside you that its (leg) gonna be cut off. You don’t know the severity of it until well down the track

(Patient participant 12/13)

I say I was in denial … you may actually start to understand what is going on but you don’t want things to go on that way

(Patient participant 20)

A lack of community awareness of acute CN made the engagement in the care process difficult for patients. Additionally, probing questions by members of the public made adapting to the treatment of their acute CN a challenge.

No matter what people say there is always bigotry out there. And people look at you … because you have this big … cast on … and then you have to go through a 10 minute spiel because no one has heard of Charcot foot

(Patient participant 16)

#### 3.1.2 Poor Access

A lack of access to the right health professional in the right place at the right time for people with acute CN was identified by health professionals and patients as a barrier to prompt CN care. Both acknowledged that a lack of timely access to appropriately skilled health professionals, multidisciplinary teams and/or diagnostic imaging delayed vital care.

I noticed swelling during the day and then it would go down.… it stayed for a about a week.… I went to the doctor … the foot still wouldn't go down. And then I couldn't get into podiatry or it was a long wait

(Patient participant 1)

Financial constraints relating to the uptake of appropriate offloading mechanisms and impaired capacity to attend appointments were identified by the health professional groups as a barrier to care.

People are declining the gold standard technique TCC … it if they don’t have the funds … (or) … they don’t have the means to come to the appointment in the first place then they think twice about it

(Podiatry participant 5)

For the patients, the inability to afford private services resulting in a reliance on publicly funded alternatives were identified as barriers to the care of people with acute CN.

… he said that I needed … I explained to him I didn’t have the type of health insurance or the funds … he said you … have to go through the public system

(Patient participant 16)

#### 3.1.3 Burden of Charcot Management

Engagement with the acute CN management process by the patient was viewed from differing perspectives by the health professionals and patients. Health professionals felt that patients did not prioritise the requirements of the treatment process.

… tell them to have a TCC or this is a long treatment process and at the end we need to give you these hundred dollar shoes and expensive devices. They’re going to be like, well, “Why can’t I go back to my Kmart shoes?”

(Prosthetist/Orthotist participant 3)

However, the patients emphasised the negative effect on quality of life and the immense mental and physical impact the prescribed treatment process took over an extended period of time. This may be due to the immobilisation required of acute CN management impacting the ability to drive, maintain employment, housing accessibility and socialisation with family and friends (Table 3).

It changed my quality of life because I was an active person … all of a sudden I couldn’t go for walks with my family

(Patient participant 20)

### 3.2 Theme 2. Enablers to Evidence‐Based Assessment, Diagnosis and Management of Acute CN

#### 3.2.1 Timely Access to Skilled and Knowledgeable Clinicians and Resources for Charcot Management

Time was identified by health professionals as the most important enabler to the care of people with acute CN and was also an important enabler for patients. When referral pathways worked and timely access to a skilled multidisciplinary team was achieved, the health professionals believed that this would support with better outcomes for people with acute CN.

A quality referral …. if we get a referral from the GP … it says: got a patient here HBA 1C of 14, swollen foot we are gonna get them in straight away

(Orthopaedic surgeon participant 1)

Timely initial diagnosis by, and access to, the HRFC team was important to the patients when they were having a clinical issue during the treatment process.

Anytime there was an issue you guys (said) don't hesitate to call … if I did have a problem your team … basically said, 'can you come down tomorrow?

(Patient participant 4)

The experience of the HRFC team was recognised by both the health professionals and people with acute CN as key to the prompt identification of acute CN. People with acute CN stated that they had trust in the HRFC team who provided education with sensitivity and clarity, which engendered confidence in information provided (Table 3).

The plaster I knew had to be done … (the podiatrist) told me … this is the average (length of time in a cast) and she told me how long I would be going … Had I not known (what to expect) I would have been busting but I knew

(Patient participant 7)

The health professionals felt that access to aids and home modifications were important to compliment the Charcot management process. Reduced wait to access funding for prescribed offloading was identified by people with acute CN as benefiting the overall care process.

Getting Physio and OT involved to make sure the house is set up to be able to use a TCC and a CAM boot …. And then wound care if there are any wounds as well

(Podiatrist participant 1)

The clear transition between the various management stages of tackling acute CN were identified by health professionals and patients as important to life‐long living with the condition. Strategies, such as strengthening of the legs, would help reduce apprehension with transition out of the supportive devices into shoes.

I feel that the majority of people we see don’t fit into the norm of … cast … CROW and then to shoes.… but when the (patient) variables change … (the team) rely heavily on the podiatrists, doctors to have that round table discussion with the patient about that timing

(Prosthetist/Orthotist participant 3)

Into the gym to strengthen up the muscles in that leg because when I first got the boot off I had to use a walking stick as I couldn’t put any pressure through that side because there was no muscles

(Patient participant 15)

### 3.3 Theme 3. Mitigating Factors to the Negative Impact of Charcot Management

Mitigating factors are considered those within the control of the health professionals and people with acute CN. They emerged during the thematic analysis as factors that reduced the negative impact of living with acute CN. The health professionals identified that the skill of the multidisciplinary team in supporting the person with acute CN was vital.

… with the multidisciplinary approach, you and your department being able to provide easy access regular review … they (are) gonna get a better outcome as a result of that

(Orthopaedic surgeon participant 3)

People with acute CN demonstrated resilience as they adapted to this significant change in lifestyle during the treatment process. The motivation to work through the process with acute CN and rehabilitate mind and body to live their best life came through in the patient interviews.

I’ve learnt to change my whole life … I’ve learnt what I can and can’t do and I probably understand more about my feet

(Patient participant 16)

Patient trust in the treating team through empathy and education was highlighted as aiding the process.

you’ve just got to put your faith in your team and say you know they know what they are talking about

(Patient participant 19)

The health professionals had identified that clearly explaining to the patient their condition and then exploring with them the options for offloading with respect to their activities daily living mitigated the negative impact of acute CN in the management process.

Seeing the X‐rays showing what the foot structure looks like as compared to what it should look like

(Prosthetist/Orthotist participant 3)

The patients highlighted the importance of being involved in the decision‐making about the type and appearance of the offloading as well as being able to maintain meaningful employment whilst receiving care for their acute CN.

I could work … with (my employer) when I had the black boot on. I wasn’t allowed to when I was in plaster

(Patient participant 7)

### 3.4 Theme 4. Suggestions for Improvement in Charcot Management

Suggestions for future consideration in the care of the patient with acute CN centred around upskilling health professionals to improve patient access to timely CN care, diabetes screening and patient‐centred CN education as well as community awareness campaigns about CN.

Probably doctor led … with diabetes educator, … access to) … pathology for monitoring kidneys. Someone teaching them (patients) about foot care what to look out for …

(Orthopaedic surgeon participant 1)

People with acute CN stated that targeted awareness campaigns and education to increase community knowledge of acute CN and its devastating impact are required.

Some people use pamphlets, some people listen to the radio. Most people are on their phones on some platform. You could do it (Charcot education campaign) on … Facebook or other thing

(Patient participant 10)

## 4 Discussion

Four overarching themes were identified: (i) barriers to the evidence‐based assessment, diagnosis and management of acute CN with subthemes of (a) inadequate knowledge and insight, (b) poor access and (c) burden of Charcot management; (ii) enablers to the evidence‐based assessment, diagnosis and management of acute CN with one subtheme (a) timely access to skilled clinicians, knowledge and resources; (iii) mitigating factors to the negative impact of Charcot management and (iv) suggestions for strategies to improve the care of acute CN.

Both health professionals and patients reported delays in identification and misdiagnosis of acute CN by health professionals and inadequate patient knowledge and understanding of acute CN as key barriers to best practice care. These findings are consistent with previous research, which suggests that people who develop acute CN are often unaware that they have the condition [6, 8, 20], and that once a person with acute CN presents to a health professional, misdiagnosis can occur due a lack of knowledge and understanding of the condition [16, 21, 22, 23]. A delay in the identification and care of acute CN may result in foot structural change, breaches of skin integrity, infection and lower limb amputation [5].

Both health professionals and patients also reported that a lack of access to the ‘right health professionals, in the right place at the right time’ was a barrier to access to timely care. Referral processes in the region were a barrier to accessing timely care, impacting appropriate triage, diagnosis and implementation of gold standard therapy. On the flipside, timely access to skilled clinicians' knowledge and resources was identified as a key enabler in the assessment diagnosis and management of acute CN. Patients made clear that the diagnosis of, and information about, acute CN was lacking until they accessed the HRFC. This is unsurprising as staff in the HRFC are specifically trained in the assessment, diagnosis and management of acute CN. Indeed, the retrospective audit of the service showed that once a patient attended the HRFC, diagnosis and gold standard treatment was immediately implemented [11].

A lack of access to appropriate diagnostic imaging was reported as a barrier to accessing care, particularly in smaller regional or rural towns. Health professionals in this study highlighted that there was no MRI in smaller towns in the Grampians region; a concern, as MRI is the diagnostic gold standard imaging for diagnosis of acute CN [2, 5, 16, 24]. The availability of TCC therapy is also limited to the HRFC in the region [11]. This barrier to TCC is serious for those unable to attend the HRFC in particular (a key finding of the retrospective audit [11]) as immediate TCC therapy is the gold standard therapy for acute CN [10]. A possible workforce initiative to overcome this geographical disparity in accessing care would be to upskill podiatrists in a regional or rural health settings to implement TCC therapy for people with acute CN [25].

The significant burden of living with acute CN was recognised as a barrier again by both the health professionals and patients. The management of acute CN can result in a reduction of quality of life of patients particularly relating to the physical health and wellbeing [4, 14], and the patients especially reinforced the mental and physical effects on their quality of life. To reduce the influence of this burden, the patients identified that where empathy, clear education and support was demonstrated by the clinicians, trust and a greater connection with the treating team could be established.

Mitigating factors to the negative impact of Charcot management was identified as a key theme, focusing on factors that were within the control of the health professional and patient. For example, patients reported more resilience and a capacity for self‐expression in decision‐making around the management of acute CN when they were actively engaged with the treating team. This could assist the patient with CN navigate the negative impacts of their quality of life demonstrated in a recent Australian study [4] such as a lack of independence and feelings of anxiety. The support of the multidisciplinary team and from respective workplaces in some instances allowed the patient to maintain meaningful employment and greater financial security.

Suggestions by the health professionals and patients were made to assist with the improvement of the assessment diagnosis and management of acute CN. Clear and accurate education provided by skilled clinicians early in the acute CN process was identified by patients to increase patient knowledge and insight into what to expect and understand the seriousness of acute CN, enabling best practice care. Health professionals reported that patient knowledge and insight into their condition of acute CN was increased if they were involved in the treatment process and decision‐making. Targeted approaches on how to deliver education relevant for patients with acute CN are not well documented in the evidence [2, 5, 16], but health professionals would benefit from guidance to increase uniformity on the messages provided to patients with acute CN.

### 4.1 Implications for Practice

Previous studies have demonstrated that the provision of evidence‐based care of acute CN in regional/rural health services is challenging [11, 22]. Although international clinical guidelines exist, it is clear from this study that there are likely to be factors that are influential in the successful delivery of gold standard care not described in the guidelines [5]. The findings from this study suggests that regional/rural areas should focus on improving knowledge of acute CN in the community, upskilling health professionals and patient support.

As indicated by the patients in this study, improving knowledge of acute CN could involve general health awareness campaigns to better disseminate information about the existence of acute CN, what to look for and when to seek help. Greater awareness of acute CN from people with diabetes and their health providers may then generate better strategies to support people with acute CN through the care process.

Specific upskilling of health professionals in regional or rural areas is crucial across both public and private health sectors. People with acute CN can present for care across a broad range of health services and professionals. Upskilling should focus on ensuring primary care providers understand the seriousness of acute CN and understand the positive impact that appropriate urgent referral has on timely access to care. As an example, the health professionals in this study identified earlier screening by diabetes educators and subsequent tailored education could facilitate early identification of acute CN and referral for gold standard care in a HRFC. Although there has been some success in upskilling rural podiatrists to implement TCC therapy in more rural areas, health service capacity—such as workforce shortages, limited podiatry availability and challenges in upskilling non‐foot‐specialist health professionals—can hinder efforts to build interdisciplinary expertise [26]. Remote monitoring by health professionals facilitated by telehealth could be a solution. Emerging evidence suggests that telehealth approaches to the management of diabetes‐related foot disease enable flexible, evidence‐based interdisciplinary management in rural and remote regions [26].

The themes identified consider patient support. Mental health decline through social isolation and diminished quality of life can take effect during the care pathway of acute CN [14, 27]. Patients felt that psychological support may be beneficial to work through mental health issues during the treatment process. Health professionals suggested person‐centred approaches to working with patients such as motivational interviewing. Embedding psychology and motivational interviewing support may assist the patient to develop strategies earlier in their Acute CN care to support their lifestyle and occupation.

### 4.2 Strengths and Limitations

A strength of this study was that it investigated the perceptions of both health professionals and patients in care of acute CN. Although a recent Australian study has provided qualitative information from people with acute CN which overlaps and compliments the results presented in this study [4], this study reports data from both patients with acute CN and health professionals. To the authors' knowledge, this is the first such study set in a regional or rural Australian setting.

Although data saturation was achieved from the small number of health professional and patient participants, it is likely that the findings do not reflect the experiences of all those with acute CN across the whole catchment region of this study and the results may not be transferrable across all settings in Australia, especially metropolitan and remote settings. The health professionals recruited were focused on secondary and tertiary care, with limited representation from the primary care sector such as general practice (who may be the first point of contact by someone who has acute CN). General practitioners or others in the primary care sector may have provided a different perspective to those recruited in the study.

The principal researcher is a staff member alongside the health professionals who participated in the focus groups and a senior clinician working with the patients. This ‘insider’ scenario could have resulted in a power imbalance and therefore the types of answers given by the health professionals and patients [28]. A pragmatic transparent approach to this research was undertaken to mitigate the principal researcher's positionality and avoid undue bias and effect on the data presented [29].

### 4.3 Conclusion

This study highlights barriers to acute CN care such as delayed diagnosis, misidentification and limited access to skilled clinicians and diagnostic tools. Timely access to knowledgeable healthcare providers, patient engagement and empathetic communication may improve CN care outcomes. Support from multidisciplinary teams and regional health providers also helps patients maintain stability during treatment. To enhance outcomes, the findings suggest that increasing awareness of CN, upskilling healthcare professionals and integrating patient‐centred and psychological support. These strategies aim to ensure timely diagnosis, equitable care and improved quality of life for those with CN.

## Author Contributions

**Dimitri Diacogiorgis:** conceptualisation, methodology, investigation, formal analysis, writing – original draft, writing – review and editing. **Byron M. Perrin:** conceptualisation, methodology, supervision, writing – review and editing. **Emma MacDonald:** formal analysis, writing – review and editing. **Michael I. C. Kingsley:** conceptualisation, methodology, supervision, writing – review and editing.

## Ethics Statement

Ethics approval was obtained through the Grampians Health and La Trobe University Human Research Ethics Committees. All participants provided written informed consent prior to participation in the study.

## Consent

The authors have nothing to report.

## Conflicts of Interest

The authors declare no conflicts of interest.

## Data Availability

All data generated and/or analysed are included in this published article.
